# Anticonvulsant Potential of Certain New (2*E*)-2-[1-Aryl-3-(1*H*-imidazol-1-yl)propylidene]-*N*-(aryl/H)hydrazinecarboxamides

**DOI:** 10.1155/2014/357403

**Published:** 2014-01-12

**Authors:** Mohamed I. Attia, Mohamed N. Aboul-Enein, Aida A. El-Azzouny, Yousreya A. Maklad, Hazem A. Ghabbour

**Affiliations:** ^1^Department of Pharmaceutical Chemistry, College of Pharmacy, King Saud University, P.O. Box 2457, Riyadh 11451, Saudi Arabia; ^2^Pharmaceutical and Drug Industries Research Division, Medicinal and Pharmaceutical Chemistry Department (Medicinal Chemistry Group), National Research Centre, Dokki, Giza 12622, Egypt; ^3^Pharmaceutical and Drug Industries Research Division, Medicinal and Pharmaceutical Chemistry Department (Pharmacology Group), National Research Centre, Dokki, Giza 12622, Egypt

## Abstract

Anticonvulsant potential and neurotoxicity of certain new imidazole-containing arylsemicarbazones **6a–p** are reported. The test compounds **6a–p** exhibited anticonvulsant activity mainly in the scPTZ screen. Compound **6p** emerged as the most active surrogate displaying 100% protection at a dose level of 636 **μ**mol/kg in the scPTZ screen without any neurotoxicity. The assigned (*E*)-configuration of the title compounds **6a–p** was confirmed *via* single crystal X-ray structure of compound **6g**.

## 1. Introduction

Epilepsy is one of the most common neurological disorders affecting nearly 50 million of mankind with the majority of cases being in developing countries [1]. Despite the availability of many new antiepileptic drugs (AEDs) and the remarkable strides in this research field, estimates suggest that about 20–30% of patients have seizures that are resistant to the available antiepileptic medications [2]. The currently approved antiepileptics have dose-related adverse effects such as ataxia, hepatotoxicity, gingival hyperplasia, and megaloblastic anaemia [3–5]. Therefore, the development of newer, more effective, and more selective antiepileptic agents with lesser side effects remains a major focus of antiepileptic drug research.

**Figure 1:**
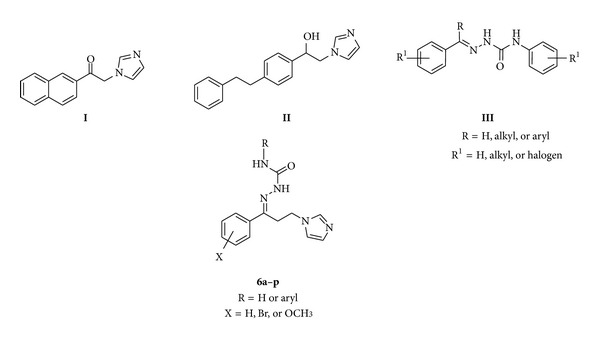
Structures of nafimidone (**I**), denzimol (**II**), arylsemicarbazones **III**, and the title compounds **6a**–**p**.

An examination of the literature revealed that aralkylimidazoles, namely, nafimidone (**I**) and danzimol (**II**) (Figure 1), exhibited anticonvulsant activity. Compounds **I** and **II** are structurally unrelated to the currently available anticonvulsants and they are independently discovered. Additionally, they showed anticonvulsant profile similar to that of phenytoin or carbamazepine while they are more potent than barbiturates and valproic acid [6–9].

Arylsemicarbazones featuring the general structural skeleton **III** (Figure 1) have been recognized as a promising pharmacophore for anticonvulsants and a sizable number of arylsemicarbazone derivatives showed anticonvulsant activity [10, 11].

In a search for new anticonvulsant chemical entities, we report herein the synthesis and characterization of certain new imidazole-containing arylsemicarbazones **6a–p** as hybrid structures incorporating both imidazole and arylsemicarbazone moieties in order to gain insight into their anticonvulsant potential (phase I) as well as their neurotoxicity. The importance of the presence of a primary amide group for the anticonvulsant activity of the target compounds **6a–p** was also explored. Additionally, the configuration of the title compounds **6a–p** around the imine double bond was examined *via* single crystal X-ray crystallography of compound **6g**.

## 2. Experimental

All melting points were determined using Electrothermal Capillary melting point apparatus and are uncorrected. Infrared (IR) spectra were recorded as KBr pellets with JASCO FT/IR-6100 spectrometer and values are represented in cm^−1^. ^1^H NMR (500 MHz) and ^13^C NMR (125 MHz) spectra were carried out on Jeol ECA 500 MHz spectrometer using TMS as an internal standard and chemical shift values were recorded in ppm on *δ* scale. The ^1^H NMR data were represented as follows: chemical shifts, multiplicity (s; singlet, d; doublet, dd; doublet of doublet, t; triplet, m; multiplet and br; broad), number of protons, and type of protons. The ^13^C NMR data were represented as chemical shifts and type of carbons. Mass spectra were measured on Agilent Triple Quadrupole 6410 QQQ LC/MS with ESI (electrospray ionization) source. Silica gel TLC (thin layer chromatography) cards from Merck (silica gel precoated aluminium cards with fluorescent indicator at 254 nm) were used for thin layer chromatography. Visualization was performed by illumination with UV light source (254 nm). The X-ray diffraction measurements of compound **6g** were performed using Bruker SMART APEXII CCD diffractometer.

### 2.1. Synthesis

#### 2.1.1. General Procedure for the Synthesis of Arylsemicarbazides **3a–f** [12]

A solution of the appropriate aniline derivative **1a–f** (20 mmol) in CH_2_Cl_2_ (20 mL) was added dropwise to a stirred solution of ethyl chloroformate (10 mmol) in CH_2_Cl_2_ (5 mL). The reaction mixture was stirred at room temperature for 0.5 hr (**2a**, **2b**, and **2d–f**) and 24 hrs (for **2c**). After completion of the reaction, the reaction mixture was filtered and the filtrate was washed with 1N HCl, dried (Na_2_SO_4_), and evaporated under reduced pressure to give the respective crude carbamates **2a–f**. A mixture containing the appropriate carbamate **2a–f** (10 mmol) and hydrazine hydrate (10 mL) was heated to reflux for 1 hr (**3f**), 2 hrs (**3b**), and 24 hrs (**3a**,** 3c–e**). The reaction mixture was cooled and filtered to yield the corresponding crude arylsemicarbazides **3a–f**. The crude products were used as such for the next reactions.

#### 2.1.2. General Procedure for Preparation of the Ketones **5a–c**

The appropriate acetophenone derivative **4a–c** (200 mmol), dimethylamine hydrochloride (270 mmol), and paraformaldehyde (90 mmol) were heated to reflux in absolute ethanol (35 mL) in the presence of catalytical amount of concentrated hydrochloric acid (0.5 mL). Reflux of the reaction mixture was continued under stirring for two hours, cooled and acetone (200 mL) was added. The formed Mannich bases were precipitated, filtered off, and dried. Subsequently, Mannich bases (100 mmol) were dissolved in water (100 mL) and imidazole (200 mmol) was added. The reaction mixture was heated to reflux for five hours, cooled and the precipitated solids were collected by filtration to give ketones **5a–c** which were pure enough to be used in the next step.

*(1) 3-(1H-Imidazol-1-yl)-1-phenylpropan-1-one* (**5a**). Synthesis of** 5a **was previously reported [13].

*(2) 1-(4-Bromorophenyl)-3-(1H-imidazol-1-yl)propan-1-one* (**5b**). Synthesis of** 5b **was previously reported [14].

*(3) 3-(1H-Imidazol-1-yl)-1-(4-methoxyphenyl)propan-1-one* (**5c**). Synthesis of** 5c **was previously reported [15].

#### 2.1.3. General Procedure for the Synthesis of the Target Compounds **6a–f**, **6h–k**, and **6m–p**

A solution containing the appropriate arylsemicarbazide **3a–f** (10 mmol), appropriate ketone **5a–c** (10 mmol), and few drops of glacial acetic acid in ethanol (15 mL) was stirred at room temperature for 18 hrs. The reaction mixture was rotovapped and the residue was crystallized from ethanol to give the title compounds **6a–f**, **6h–k**, and **6m–p**.

*(1) (2E)-2-[3-(1H-Imidazol-1-yl)-1-phenylpropylidene]-N-phenylhydrazinecarboxamide* (**6a**). Synthesis and characterization of **6a** were recently reported [16].

*(2) (2E)-N-(4-Bromophenyl)-2-[3-(1H-imidazol-1-yl)-1-phenylpropylidene]hydrazinecarboxamide* (**6b**). Yield 60%; white solid m.p. 198–200°C; **ν** (cm^−1^) 3440, 3200 (NH), 1664 (C=O), 1585 (C=N); ^1^H NMR (DMSO-*d*_6_): *δ* 3.33 (t, *J* = 7.7 Hz, 2H, –C*H*_*2*_–CH_2_–N), 4.08 (t, *J* = 7.6 Hz, 2H, –CH_2_–C*H*_*2*_–N), 6.81 (s, 1H, –N–C*H*=CH–N=), 7.23 (s, 1H, –N–CH=C*H*–N=), 7.35-7.36 (m, 3H, Ar–H), 7.43 (d, *J* = 8.4 Hz, 2H, Ar–H), 7.59–7.62 (m, 3H, Ar–H, –N–C*H*=N–), 7.79–7.81 (m, 2H, Ar–H), 8.79 (s, 1H, NH), 10.29 (s, 1H, NH); ^13^C NMR (DMSO-*d*_6_): *δ* 28.8 (–*C*H_2_–CH_2_–N), 42.7 (–CH_2_–*C*H_2_–N), 114.6 (Ar–C), 119.9 (–N–*C*H=CH–N=), 122.1, 127.2, 128.6, 128.8, 129.7, 131.8 (–N–CH=*C*H–N=, Ar–CH), 137.2, 137.9, 139.3 (–N–CH=N–, Ar–C), 145.9 (C=O), 154.1 (C=N); MS *m*/*z* (ESI): 412.1 [M]^+^.

*(3) (2E)-N-(4-Chlorophenyl)-2-[3-(1H-imidazol-1-yl)-1-phenylpropylidene]hydrazinecarboxamide* (**6c**). Yield 65%; white solid m.p. 204–206°C; **ν** (cm^−1^) 3491, 3199 (NH), 1665 (C=O), 1589 (C=N); ^1^H NMR (DMSO-*d*_6_): *δ* 3.33 (t, *J* = 7.5 Hz, 2H, –C*H*_*2*_–CH_2_–N), 4.08 (t, *J* = 7.4 Hz, 2H, –CH_2_–C*H*_*2*_–N), 6.82 (s, 1H, –N–C*H*=CH–N=), 7.24 (s, 1H, –N–CH=C*H*–N=), 7.31–7.39 (m, 5H, Ar–H), 7.59 (s, 1H, –N–C*H*=N–), 7.62–7.67 (m, 2H, Ar–H), 7.80–7.82 (m, 2H, Ar–H), 8.99 (s, 1H, NH), 10.29 (s, 1H, NH); ^13^C NMR (DMSO-*d*_6_): *δ* 28.8 (–*C*H_2_–CH_2_–N), 40.2 (–CH_2_–*C*H_2_–N), 120.0 (–N–C*H*=CH–N=), 122.1, 126.8, 127.0, 128.7, 128.8, 128.9, 129.5 (–N–CH=*C*H–N=, Ar–CH, Ar–C), 137.2, 137.9, 138.5 (–N–CH=N–, Ar–C), 145.9 (C=O), 154.1 (C=N); MS *m*/*z* (ESI): 368.21 [M + 1]^+^.

*(4) (2E)-2-[3-(1H-Imidazol-1-yl)-1-phenylpropylidene]-N-(2-methylphenyl)hydrazinecarboxamide* (**6d**) [17]. Yield 54%; pale yellow solid m.p. 188–190°C; IR (KBr): **ν** (cm^−1^) 3649, 3385 (NH), 1700 (C=O), 1588 (C=N); ^1^H NMR (DMSO-*d*_6_): *δ* 2.24 (s, 3H, CH_3_), 3.33 (t, *J* = 7.6 Hz, 2H, –C*H*_*2*_–CH_2_–N), 4.09 (t, *J* = 7.5 Hz, 2H, –CH_2_–C*H*_*2*_–N), 6.82 (s, 1H, –N–C*H*=CH–N=), 6.94–7.01 (m, 1H, Ar–H), 7.13–7.23 (m, 2H, Ar–H), 7.25 (s, 1H, –N–CH=C*H*–N=), 7.32–7.39 (m, 3H, Ar–H), 7.61 (s, 1H, –N–C*H*=N–), 7.72–7.83 (m, 3H, Ar–H), 8.58 (s, 1H, NH), 10.33 (s, 1H, NH); ^13^C NMR (DMSO-*d*_6_): *δ* 17.9 (CH_3_), 28.9 (–*C*H_2_–CH_2_–N), 40.3 (–CH_2_–*C*H_2_–N), 120.0 (–N–*C*H=CH–N=), 122.6, 124.2, 126.7, 126.8, 128.8, 129.0, 129.5, 129.6, 130.7 (–N–CH=*C*H–N=, Ar–CH, Ar–C), 136.5, 137.3, 137.9 (Ar–C), 137.3 (–N–CH=N–), 145.3 (C=O), 154.1 (C=N); MS *m*/*z* (ESI): 348.3 [M + 1]^+^.

*(5) (2E)-2-[3-(1H-Imidazol-1-yl)-1-phenylpropylidene]-N-(4-methylphenyl)hydrazinecarboxamide* (**6e**). Yield 48%; white solid m.p. 203–205°C; IR (KBr): **ν** (cm^−1^) 3440, 3209 (NH), 1657 (C=O), 1593 (C=N); ^1^H NMR (DMSO-*d*_6_): *δ* 2.28 (s, 3H, CH_3_), 3.33 (t, *J* = 7.7 Hz, 2H, –C*H*_*2*_–CH_2_–N), 4.14 (t, *J* = 7.4 Hz, 2H, –CH_2_–C*H*_*2*_–N), 6.87 (s, 1H, –N–C*H*=C*H*–N=), 7.13 (d, *J* = 8.2 Hz, 2H, Ar–H), 7.29 (s, 1H, –N–CH=C*H*–N=), 7.39–7.43 (m, 3H, Ar–H), 7.53 (d, *J* = 8.2 Hz, 2H, Ar–H), 7.65 (s, 1H, –N–CH=N–), 7.84–7.86 (m, 2H, Ar–H), 8.79 (s, 1H, NH), 10.24 (s, 1H, NH); ^13^C NMR (DMSO-*d*_6_): *δ* 20.4 (CH_3_), 28.3 (–*C*H_2_–CH_2_–N), 42.1 (–CH_2_–*C*H_2_–N), 119.4 (–N–*C*H=CH–N=), 120.0, 126.4, 128.3, 128.4, 128.8, 128.9 (–N–CH=*C*H–N=, Ar–CH), 131.5, 136.3, 136.8 (Ar–C), 137.3 (–N–CH=N–), 144.9 (C=O), 153.6 (C=N); MS *m*/*z* (ESI): 348.2 [M + 1]^+^.

*(6) (2E)-N-(2,4-Dichlorophenyl)-2-[3-(1H-imidazol-1-yl)-1-phenylpropylidene]hydrazinecarboxamide* (**6f**). Yield 69%; white solid m.p. 198–200°C; IR (KBr): **ν** (cm^−1^) 3396, 3109 (NH), 1706 (C=O), 1578 (C=N); ^1^H NMR (DMSO-*d*_6_): *δ* 3.32 (t, *J* = 7.5 Hz, 2H, –C*H*_*2*_–CH_2_–N), 4.09 (t, *J* = 7.6 Hz, 2H, –C*H*_*2*_–CH_2_–N), 6.81 (s, 1H, –N–C*H*=CH–N=), 7.23 (s, 1H, –N–CH=C*H*–N=), 7.37–7.39 (m, 4H, Ar–H), 7.59 (s, 1H, –N–CH=N–), 8.65 (d, *J* = 2.1 Hz, 1H, Ar–H), 7.72 (d, *J* = 6.9 Hz, 2H, Ar–H), 8.24 (d, *J* = 8.4 Hz, 1H, Ar–H), 9.11 (s, 1H, NH), 10.72 (s, 1H, NH); ^13^C NMR (DMSO-*d*_6_): *δ* 29.1 (–*C*H_2_–CH_2_–N), 40.2 (–CH_2_–*C*H_2_–N), 119.9 (–N–*C*H=CH–N=), 122.0, 123.7, 126.6, 127.2, 128.5, 128.8, 128.9, 129.2, 129.8, (–N–CH=*C*H–N=, Ar–CH, Ar–C), 134.9, 136.9, 137.9 (–N–CH=N–, Ar–C), 146.6 (C=O), 153.3 (C=N); MS *m*/*z* (ESI): 402.1 [M]^+^.

*(7) (2E)-2-[1-(4-Bromophenyl)-3-(1H-imidazol-1-yl) propylidene]-N-(4-chlorophenyl)hydrazinecarboxamide (* **6h**). Yield 35%; pale yellow solid m.p. 200–202°C; **ν** (cm^−1^) 3367, 3197 (NH), 1666 (C=O), 1591 (C=N); ^1^H NMR (DMSO-*d*_6_): *δ* 3.26 (t, *J* = 7.7 Hz, 2H, –C*H*_*2*_–CH_2_–N), 4.06 (t, *J* = 7.7 Hz, 2H, –CH_2_–C*H*_*2*_–N), 6.80 (s, 1H, –N–C*H*=CH–N=), 7.22 (s, 1H, –N–CH=C*H*–N=), 7.32 (d, *J* = 9.2 Hz, 2H, Ar–H), 7.51 (d, *J* = 9.2 Hz, 2H, Ar–H), 7.57 (s, 1H, –N–CH=N–), 7.65 (d, *J* = 8.4 Hz, 2H, Ar–H), 7.76 (d, *J* = 8.4 Hz, 2H, Ar–H), 8.99 (s, 1H, NH), 10.33 (s, 1H, NH); ^13^C NMR (DMSO-*d*_6_): *δ* 28.6 (–*C*H_2_–CH_2_–N), 40.2 (–CH_2_–*C*H_2_–N), 119.9 (–N–*C*H=CH–N=), 122.2, 122.9, 126.8, 128.9, 129.1 (–N–CH=*C*H–N=, Ar–CH), 131.7, 136.5, 137.9, 138.5 (–N–CH=N–, Ar–C), 144.9 (C=O), 154.0 (C=N); MS *m*/*z* (ESI): 446.1 [M]^+^.

*(8) *(2*E*)-*2-[1-(4-Bromophenyl)-3-(1H-imidazol-1-yl)propylidene]-N-(2-methylphenyl)hydrazinecarboxamide* (**6i**). Yield 45%; white solid m.p. 200–202°C; 3485, 3379 (NH), 1688 (C=O), 1587 (C=N); ^1^H NMR (DMSO-*d*_6_): *δ* 2.27 (s, 3H, CH_3_), 3.33 (br. s, 2H, –C*H*_*2*_–CH_2_–N), 4.12 (br. s, 2H, –CH_2_–C*H*_*2*_–N), 6.85 (s, 1H, –N–C*H*=CH–N=), 7.05–7.09 (m, 1H, Ar–H), 7.20–7.23 (m, 2H, Ar–H), 7.26 (s, 1H, –N–CH=C*H*–N=), 7.55–7.57 (m, 2H, Ar–H), 7.63 (s, 1H, –N–CH=N–), 7.73–7.77 (m, 3H, Ar–H), 8.60 (s, 1H, NH), 10.40 (s, 1H, NH); ^13^C NMR (DMSO-*d*_6_): *δ* 18.0 (CH_3_), 28.6 (–C*H*_*2*_–CH_2_–N), 42.6 (–CH_2_–C*H*_*2*_–N), 120.0 (–N–*C*H=CH–N=), 122.8, 123.3, 124.4, 126.7, 128.8, 130.3, 130.7 (–N–CH=*C*H–N=, Ar–CH), 131.8, 136.6, 137.2, 137.9 (–N–CH=N–, Ar–C), 144.3 (C=O), 154.1 (C=N); MS *m*/*z* (ESI): 426.2 [M]^+^. 

*(9) (2E)-2-[1-(4-Bromophenyl)-3-(1H-imidazol-1-yl) propylidene]-N-(4-methylphenyl)hydrazinecarboxamide* (**6j**). Yield 35%; pale yellow solid m.p. 204–206°C; **ν** (cm^−1^) 3450, 3195 (NH), 1656 (C=O), 1594 (C=N); ^1^H NMR (DMSO-*d*_6_): *δ* 2.22 (s, 3H, CH_3_), 3.23 (t, *J* = 7.7 Hz, 2H, –C*H*_*2*_–CH_2_–N), 4.06 (t, *J* = 7.7 Hz, 2H, –C*H*_*2*_–CH_2_–N), 6.85 (s, 1H, –N–C*H*=CH–N=), 7.07 (d, *J* = 8.4 Hz, 2H, Ar–H), 7.21 (s, 1H, –N–CH=*C*H–N=), 7.45 (d, *J* = 8.4 Hz, 2H, Ar–H), 7.51 (d, *J* = 9.2 Hz, 2H, Ar–H), 7.57 (s, 1H, –N–CH=N–), 7.74 (d, *J* = 8.4 Hz, 2H, Ar–H), 8.76 (s, 1H, NH), 10.20 (s, 1H, NH); ^13^C NMR (DMSO-*d*_6_): *δ* 20.9 (CH_3_), 28.5 (–*C*H_2_–CH_2_–N), 42.6 (–CH_2_–*C*H_2_–N), 120.0 (–N–*C*H=CH–N=), 120.7, 122.8, 128.9, 129.5, 131.7 (–N–CH=*C*H–N=, Ar–CH), 132.2, 136.5, 136.7, 137.9 (–N–CH=N–, Ar–C), 144.4 (C=O), 154.0 (C=N); MS *m*/*z* (ESI): 426.2 [M]^+^.

*(10) (2E)-2-[1-(4-Bromophenyl)-3-(1H-imidazol-1-yl) propylidene]-N-(2,4-dichlorophenyl)hydrazinecarboxamide* (**6k**). Yield 59%; off white solid m.p. 220–222°C; IR (KBr): **ν** (cm^−1^) 3416, 3340 (NH), 1712 (C=O), 1578 (C=N); ^1^H NMR (DMSO-*d*_6_): *δ* 3.32 (t, *J* = 7.6 Hz, 2H, –C*H*_*2*_–CH_2_–N), 4.12 (t, *J* = 7.6 Hz, 2H, –CH_2_–C*H*_*2*_–N), 6.85 (s, 1H, –N–*C*H=CH–N=), 7.25 (s, 1H, –N–CH=C*H*–N=), 7.45 (dd, *J* = 2.4, 8.9 Hz, 1H, Ar–H), 7.59–7.61 (m, 3H, –N–CH=N–, Ar–H), 7.68–7.70 (m, 3H, Ar–H), 8.26 (d, *J* = 8.9 Hz, 1H, Ar–H), 9.09 (s, 1H, NH), 10.78 (s, 1H, NH); ^13^C NMR (DMSO-*d*_6_): *δ* 28.3 (–*C*H_2_–CH_2_–N), 42.1 (–CH_2_–*C*H_2_–N), 119.4 (–N–*C*H=CH–N=), 121.8, 122.7, 123.5, 126.8, 127.9, 128.0, 128.3, 128.6, 131.5, 134.3, 135.8 (–N–CH=*C*H–N=, Ar–CH, Ar–C), 137.3 (–N–CH=N–), 145.1 (C=O), 152.7 (C=N); MS *m*/*z* (ESI): 482.1 [M + 1]^+^. 

*(11) (2E)-N-(4-Bromophenyl)-2-[3-(1H-imidazol-1-yl)-1-(4-methoxyphenyl)propylidene]hydrazinecarboxamide* (**6m**). Yield 43%; pale yellow solid m.p. 198–200°C; 3438, 3216 (NH), 1672 (C=O), 1591 (C=N); ^1^H NMR (DMSO-*d*_6_): *δ* 3.24 (t, *J* = 7.7 Hz, 2H, –C*H*_*2*_–CH_2_–N), 3.76 (s, 3H, OCH_3_), 4.06 (t, *J* = 7.7 Hz, 2H, –CH_2_–C*H*_*2*_–N), 6.82 (s, 1H, –N–C*H*=CH–N=), 6.90 (d, *J* = 9.2 Hz, 2H, Ar–H), 7.24 (s, 1H, –N–CH=*C*H–N=), 7.44 (d, *J* = 8.4 Hz, 2H, Ar–H), 7.59 (s, 1H, –N–CH=N–), 7.62 (d, *J* = 9.2 Hz, 2H, Ar–H), 7.77 (d, *J* = 9.2 Hz, 2H, Ar–H), 8.95 (s, 1H, NH), 10.18 (s, 1H, NH); ^13^C NMR (DMSO-*d*_6_): *δ* 28.8 (–*C*H_2_–CH_2_–N), 40.4 (–CH_2_–*C*H_2_–N), 55.7 (OCH_3_), 114.2 (Ar–CH), 119.9 (–N–*C*H=CH–N=), 122.3, 128.6, 128.8, 129.7 (–N–CH=*C*H–N=, Ar–CH), 131.7, 137.9, 139.0 (–N–CH=N–, Ar–C), 145.9 (C=O), 154.1 (C=N), 160.5 (Ar–C); MS *m*/*z* (ESI): 442.2 [M]^+^.

*(12) (2E)-2-[3-(1H-Imidazol-1-yl)-1-(4-methoxyphenyl)propylidene]-N-(2-methylphenyl)hydrazinecarboxamide* (**6n**) [18]. Yield 71%; pale yellow solid m.p. 190–192°C; 3427, 3372 (NH), 1708 (C=O), 1586 (C=N); ^1^H NMR (DMSO-*d*_6_): *δ* 2.23 (s, 3H, CH_3_), 3.24 (t, *J* = 7.7 Hz, 2H, –C*H*_*2*_–CH_2_–N), 3.75 (s, 3H, OCH_3_), 4.07 (t, *J* = 7.7 Hz, 2H, –CH_2_–C*H*_*2*_–N), 6.82 (s, 1H, –N–C*H*=CH–N=), 6.90 (d, *J* = 8.4 Hz, 2H, Ar–H), 7.90 (t, *J* = 7.7 Hz, 1H, Ar–H), 7.14–7.19 (m, 2H, Ar–H), 7.22 (s, 1H, –N–CH=C*H*–N=), 7.59 (s, 1H, –N–CH=N–), 7.70 (d, *J* = 9.2 Hz, 2H, Ar–H), 7.77 (d, *J* = 7.7 Hz, 1H, Ar–H), 8.52 (s, 1H, NH), 10.16 (s, 1H, NH); ^13^C NMR (DMSO-*d*_6_): *δ* 18.0 (CH_3_), 28.8 (–*C*H_2_–CH_2_–N), 40.3 (–CH_2_–*C*H_2_–N), 55.8 (OCH_3_), 114.4 (Ar–CH), 120.0 (–N–*C*H=CH–N=), 122.4, 124.0, 126.8, 128.1, 128.8, 129.3, 129.8 (–N–CH=*C*H–N=, Ar–CH, Ar–C), 130.7, 137.3, 137.9 (–N–CH=N–, Ar–C), 145.4 (C=O), 154.2 (C=N), 160.5 (Ar–C); MS *m*/*z* (ESI): 378.3 [M + 1]^+^. 

*(13) (2E)-2-[3-(1H-Imidazol-1-yl)-1-(4-methoxyphenyl)propylidene]-N-(4-methylphenyl)hydrazinecarboxamide* (**6o**). Yield 63%; pale yellow solid m.p. 188–190°C; 3450, 3220 (NH), 1684 (C=O), 1558 (C=N); ^1^H NMR (DMSO-*d*_6_): *δ* 2.26 (s, 3H, CH_3_), 3.26 (t, *J* = 7.7 Hz, 2H, –C*H*_*2*_–CH_2_–N), 3.79 (s, 3H, OCH_3_), 4.08 (t, *J* = 7.7 Hz, 2H, –CH_2_–C*H*_*2*_–N), 6.86 (s, 1H, –N–C*H*=CH–N=), 6.94 (d, *J* = 8.4 Hz, 2H, Ar–H), 7.11 (d, *J* = 7.7 Hz, 2H, Ar–H), 7.27 (s, 1H, –N–CH=*C*H–N=), 7.50 (d, *J* = 7.7 Hz, 2H, Ar–H), 7.63 (s, 1H, –N–CH=N–), 7.79 (d, *J* = 8.4 Hz, 2H, Ar–H), 8.74 (s, 1H, NH), 10.08 (s, 1H, NH); ^13^C NMR (DMSO-*d*_6_): *δ* 20.9 (CH_3_), 28.7 (–*C*H_2_–CH_2_–N), 40.2 (–CH_2_–*C*H_2_–N), 55.7 (OCH_3_), 114.3 (Ar–CH), 119.9 (–N–*C*H=CH–N=), 128.4, 128.8, 129.5, 129.8 (–N–CH=*C*H–N=, Ar–CH), 131.9, 136.9, 137.9 (–N–CH=N–, Ar–C), 145.4 (C=O), 154.2 (C=N), 160.6 (Ar–C); MS *m*/*z* (ESI): 378.3 [M + 1]^+^.

*(14) (2E)-N-(2,4-Dichlorophenyl)-2-[3-(1H-imidazol-1-yl)-1-(4-methoxyphenyl)propylidene]hydrazinecarboxamide* (**6p**). Yield 85%; white solid m.p. 212–214°C; IR (KBr): **ν** (cm^−1^) 3501, 3343 (NH), 1708 (C=O), 1578 (C=N); ^1^H NMR (DMSO-*d*_6_): *δ* 3.29 (t, *J* = 7.4 Hz, 2H, –C*H*_*2*_–CH_2_–N), 3.81 (s, 3H, OCH_3_), 4.12 (t, *J* = 7.4 Hz, 2H, –CH_2_–C*H*_*2*_–N), 6.86 (s, 1H, –N–C*H*=CH–N=), 6.99 (d, *J* = 8.8 Hz, 2H, Ar–H), 7.27 (s, 1H, –N–CH=C*H*–N=), 7.44 (dd, *J* = 2.3, 8.9 Hz, 1H, Ar–H), 7.69 (d, *J* = 2.4 Hz, 1H, Ar–H), 7.63 (s, 1H, –N–CH=N–), 7.72 (d, *J* = 8.8 Hz, 2H, Ar–H), 8.31 (d, *J* = 8.9 Hz, 1H, Ar–H), 9.15 (s, 1H, NH), 10.63 (s, 1H, NH); ^13^C NMR (DMSO-*d*_6_): *δ* 28.5 (–*C*H_2_–CH_2_–N), 42.3 (–CH_2_–*C*H_2_–N), 55.3 (OCH_3_), 114.0 (Ar–CH), 119.4 (–N–*C*H=CH–N=), 121.3, 122.9, 126.5, 127.5, 127.9, 128.3, 128.5, 128.9, 134.4 (–N–CH=*C*H–N=, Ar–CH, Ar–C), 131.5, 136.3, 136.8 (Ar–C), 137.3 (–N–CH=N–), 146.1 (C=O), 152.8 (C=N), 160.2 (Ar–C); MS *m*/*z* (ESI): 432.4 [M]^+^.

#### 2.1.4. General Procedure for the Synthesis of the Target Compounds **6g** and **6l**

A solution containing **5a **and/or** 5b** (4.3 mmol), semicarbazide hydrochloride (4.3 mmol), and anhydrous sodium acetate (4.3 mmol) in absolute ethanol (40 mL) was stirred at room temperature for 18 hrs. The reaction mixture was filtered and the filtrate was evaporated under reduced pressure. The residue was crystallized from ethanol to give the title compounds **6k** and **6l**. 

*(1) (2E)-2-[3-(1H-Imidazol-1-yl)-1-phenylpropylidene]hydrazinecarboxamide* (**6g**). Yield 40%; white solid m.p. 138–140°C; IR (KBr): **ν** (cm^−1^) 3469 (NH), 3435, 3194 (NH_2_), 1682 (C=O), 1565 (C=N); ^1^H NMR (DMSO-*d*_6_): *δ* 3.34 (t, *J* = 7.8 Hz, 2H, –C*H*_*2*_–CH_2_–N), 4.13 (t, *J* = 7.8 Hz, 2H, –CH_2_–C*H*_*2*_–N), 6.87 (s, 1H, –N–C*H*=CH–N=), 7.03–7.06 (m, 1H, Ar–H), 7.29 (s, 1H, –N–CH=C*H*–N=), 7.31–7.34 (m, 2H, Ar–H), 7.40 (s, 2H, –NH_2_), 7.65 (s, 1H, –N–CH=N–), 7.85-7.86 (m, 2H, Ar–H), 8.89 (s, 1H, NH); ^13^C NMR (DMSO-*d*_6_): *δ* 28.3 (–*C*H_2_–CH_2_–N), 42.1 (–CH_2_–*C*H_2_–N), 119.4 (–N–*C*H=CH–N=), 119.9, 126.4, 128.4, 128.5 (–N–CH=*C*H–N=, Ar–CH), 136.8 (Ar–C), 137.3 (–N–*C*H=N–), 145.1 (C=O), 153.6 (C=N); MS *m*/*z* (ESI): 258.2 [M + 1]^+^.

*(2) (2E)-2-[1-(4-Bromophenyl)-3-(1H-imidazol-l-yl-propylidene]hydrazinecarboxamide* (**6l**). Yield 65%; pale yellow solid m.p. 198–200°C; IR (KBr): **ν** (cm^−1^) 3466 (NH), 3300, 3194 (NH_2_), 1688 (C=O), 1589 (C=N); ^1^H NMR (DMSO-*d*_6_): *δ* 3.17 (t, *J* = 6.9 Hz, 2H, –C*H*_*2*_–CH_2_–N), 4.02 (t, *J* = 6.9 Hz, 2H, –CH_2_–C*H*_*2*_–N), 6.51 (s, 2H, –NH_2_), 6.79 (s, 1H, –N–C*H*=CH–N=), 7.18 (s, 1H, –N–CH=C*H*–N=), 7.46 (d, *J* = 8.4 Hz, 2H, Ar–H), 7.55 (s, 1H, –N–CH=N–), 7.67 (d, *J* = 8.4 Hz, 2H, Ar–H), 9.79 (s, 1H, NH); ^13^C NMR (DMSO-*d*_6_): *δ* 28.1 (–*C*H_2_–CH_2_–N), 42.6 (–CH_2_–*C*H_2_–N), 119.9 (–N–*C*H=CH–N=), 122.5, 128.7, 131.7, 136.7 (–N–CH=*C*H–N=, Ar–CH, Ar–C), 137.8 (–N–CH=N–), 142.8 (C=O), 157.8 (C=N); MS *m*/*z* (ESI): 336.1 [M]^+^.

### 2.2. Anticonvulsant Screening

#### 2.2.1. Materials and Methods

*(1) Materials*. Adult male Swiss albino mice weighing 18–25 g were used in our investigations and were purchased from Animals House Colony of the National Research Centre, Cairo, Egypt. Animals were housed under standardized conditions (room temperature 23 + 2°C; relative humidity 55 + 5%; 12 hrs-light/dark cycle) and had free access to tap water and standard mice chow throughout the whole experimental period. All animal procedures were performed in accordance with the Ethics Committee of the National Research Center and in accordance with the recommendations for the proper care and use of laboratory animals “Canadian Council on Animal Care Guidelines, 1984.” After seven days of adaptation to laboratory conditions, the animals were randomly assigned to experimental groups consisting of 6 mice each. All the test compounds were suspended in 7% tween-80 saline solutions. 

*(2) Chemicals and Drugs. *Tween-80 (Sigma, St. Louis, HO, USA), pentylenetetrazole (PTZ, Sigma, St. Louis, USA), phenytoin sodium (Nasr Co., Egypt), and phenobarbitone (Memphis Co., Egypt) were used. 

*(3) Methods*

*(a) Subcutaneous Pentylenetetrazole (scPTZ) Screen. *Aqueous solution of scPTZ (85 mg/kg) was administered in a loose fold of skin on the back of the mice necks half an hour after intraperitoneal (i.p.) injection of the test compounds **6a–p** and the animals were observed during the following 0.5 hr for the occurrence of seizures. A threshold convulsion was defined as one episode of clonic convulsions which persisted for at least a 5-second period. Absence of even a single 5-second episode of clonic spasms during the period of observation is taken as the end point in this test [19]. 

*(b) Maximal Electroshock Seizure (MES) Screen. *Electroconvulsions were turned out half an hour after i.p. injection of the test compounds **6a–p**, by a current (fixed current intensity of 25 mA, 0.2 s stimulus duration) delivered *via* earclip electrodes by a Rodent Shocker Generator (constant-current stimulator Type 221, Hugo Sachs Elektronik, Freiburg, Germany). The criterion for the occurrence of seizure activity was the tonic hind limb extension (i.e., the hind limbs of animals outstretched 180° to the plane of the body axis) [20]. 

*(c) Neurotoxicity. *In this test, the animals were trained to maintain equilibrium on a rotating 1-inch-diameter knurled plastic rod at a speed of 6 rev/min for at least 1 min in each of the three trials using rotarod device (UGO Basile, 47600, Varese, Italy). Only animals that fulfill this criterion were included into the experiment. The selected trained animals were classified into groups; six mice were used in the control group as well as in the experimental groups. The animals in the experimental groups were given the test compounds **6a–p** in 7% aqueous suspension of Tween 80 *via* i.p. route; meanwhile, the control group received the vehicle. Thirty minutes later, the mice were placed again on the rotating rod and the neurotoxicity was indicated by the inability of the animal to maintain equilibrium on the rod for at least 1 min [21].

## 3. Results and Discussion

### 3.1. Chemistry

**Scheme 1:**
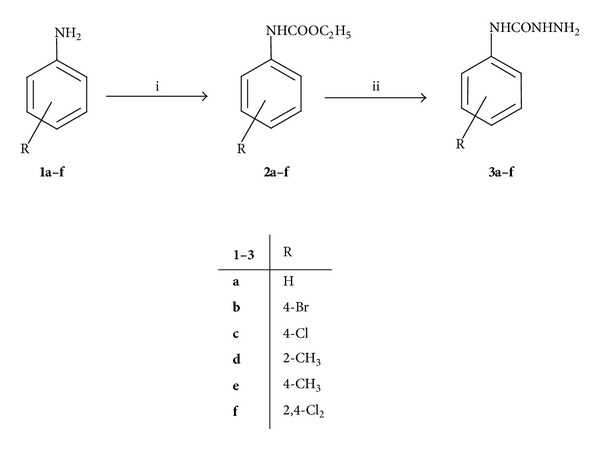
Synthetic pathway for preparation of arylsemicarbazides **3a**–**f**. Reagents and conditions: (i) ClCOOC_2_H_5_, CH_2_Cl_2_, RT, 0.5 hr; (ii) H_2_N–NH_2_·H_2_O, reflux, 24 hrs.

Synthetic strategy to synthesize arylsemicarbazides **3a–f** was previously reported [12]. Thus, the appropriate aniline derivative **1a–f** was allowed to react with ethyl chloroformate to give carbamates **2a–f**. Compounds **2a–f** were refluxed with hydrazine hydrate to yield arylsemicarbazides **3a–f** (Scheme 1).

**Scheme 2:**
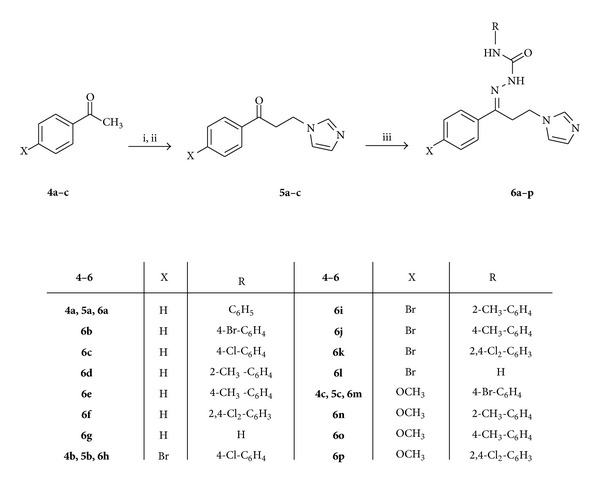
Synthetic route for preparation of the target compounds **6a**–**p**. Reagents and conditions: (i) HN(CH_3_)_2_·HCl, (CH_2_O)_*n*_, conc. HCl, ethanol, reflux, 2 hrs; (ii) imidazole, water, reflux, 5 hrs; (iii) appropriate semicarbazide **3a–f**, ethanol, acetic acid, RT, 18 hrs or semicarbazide hydrochloride, anhydrous sodium acetate, ethanol, RT, 18 hrs for **6g** and **6l**.

The target compounds **6a–p** were synthesized as portrayed in Scheme 2. The appropriate acetophenone derivative **4a–c** was transformed into the penultimate ketones **5a–c** in a two-step reaction sequence as previously reported [13]. Ketones **5a–c** were allowed to react with the appropriate arylsemicarbazide **3a–f** and/or semicarbazide hydrochloride in ethanol at room temperature to give the respective title compounds **6a–p** in moderate yields.

### 3.2. Crystal Data for Compound **6g**

**Figure 2:**
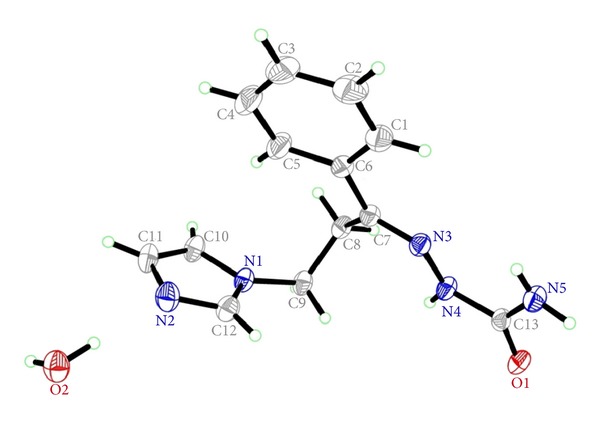
ORTEP diagram of compound **6g** drawn at 50% ellipsoids for nonhydrogen atoms.

Single crystal X-ray crystallography is a doubtless decisive analytical tool which can confirm the configuration of the imine double bond in the target compounds **6a–p**. Fortunately, we have succeeded to get single crystals of compound **6g** which were suitable for X-ray crystallography as a representative example for the synthesized target compounds **6a–p**. Accordingly, the assigned (*E*)-configuration of compound **6g** was established *via* its single crystal X-ray structure (Figure 2).

Molecular formula of **6g**: C_13_H_17_N_5_O_2_, Molecular weight: 275.32, Monoclinic, P2_1_/*c*, *a* = 11.1922 (3) Å, *b* = 5.5226 (2) Å, *c* = 22.7024 (6) Å, *β* = 99.914 (1)°, *V* = 1382.28 (7) Å^3^, *D*_calc_ = 1.323 Mg m^−3^, colorless plate with 0.92 × 0.31 × 0.06 mm. A total of 5371 reflections were measured, of which 2301 were independent. *R*_int_ = 0.031, dataset (*h*; *k*;*l*) = −13, 12; −6,4; −25,26. Refinement of *F* ^2^, against all reflections, led to *R*[*F* ^2^ > 2*σ*(*F* ^2^)] = 0.039, *wR *(*F* ^2^) = 0.130, and *S* = 0.72.

The chemical structures of the title compounds **5a–p** were confirmed *via* IR, ^1^H NMR, ^13^C NMR, and mass spectral data.

### 3.3. Anticonvulsant Activity and SAR

The preliminary anticonvulsant activity (phase I screening) of the target compounds **6a–p** was determined according to the phase I tests of the Antiepileptic Drug Development (ADD) program. ADD program was developed by National Institute of Neurological Disorders and Stroke (NINIDS) and it includes the subcutaneous pentylenetetrazole (scPTZ) screen and the maximal electroshock seizure (MES) screen [22]. scPTZ and MES screens are considered as the “gold standard” seizure model screens where they are used to identify compounds that elevate seizure threshold and to indicate the ability of the test compounds to prevent seizure spread, respectively. Additionally, acute toxicity from antiepileptic drugs in rodents is almost invariably manifested by neurological deficits. These include sedation, altered motor activity, ataxia, and impaired righting reflexes. These effects of antiepileptic drugs are often summarized by the term “neurotoxicity.” Minimal neurological deficit, such as impaired motor function, can be detected by standardized test, that is, by the rotarod test [21]. The obtained data expressed as % protection for anticonvulsant activity of the test compounds **6a–p** as well as their neurotoxicity are presented in Table 1.

Nafimidone (**I**) and danzimol (**II**) are aralkylimidazole derivatives exhibiting anticonvulsant activity. On the other hand, it has been reported that a sizable number of arylsemicarbazones show anticonvulsant activity in both scPTZ and MES screens [10, 11]. Additionally, arylsemicarbazones interact at a putative binding site *in vivo *which was designated as a hydrogen bonding area and an aryl binding site in order to exert their anticonvulsant activity. Therefore, it is likely that the pharmacophoric descriptors in arylsemicarbazones are thought to be a hydrogen bonding semicarbazone group and a lipophilic aryl ring which align at their complementary areas on the macromolecular complex *in vivo *[23]. The presence of imidazole moiety and hydrogen bonding area in the title compounds **6a–p** could improve their binding with the complementary receptor binding area *in vivo*. Accordingly, compound **6g** was synthesized as a hybrid structure containing both imidazole and arylsemicarbazone moieties to be screened as a new chemical entity with anticonvulsant potential. Compound **6g** displayed only 50% protection in the scPTZ screen at dose level of 972 *μ*mol/kg and was devoid of any activity in the MES screen.

Substitution of the aromatic ring in **6g** with a substituent endowed with a negative inductive effect like bromo-substituent gave compound **6l**. Compound **6l** showed better anticonvulsant activity than that of **6g** in both scPTZ (100% protection at a dose level of 818 *μ*mol/kg) and MES screens (16% protection at a dose level of 744 *μ*mol/kg). Additionally, the terminal hydrogen of the amide moiety of **6g** and **6l** has been replaced with aryl moiety containing substituents endowed with different electronic and steric properties in order to investigate the influence of the primary amide group on the anticonvulsant activity of this type of imidazole-containing arylsemicarbazones. Accordingly, compounds **6a–f**, **6i–k**, and **6m–p** were synthesized and screened for their anticonvulsant potential. In compounds **6a–f**, compound **6f**, bearing 2,4-dichloro substituents, is the most active candidate having 100% protection in the scPTZ screen at a dose level of 684 *μ*mol/kg without any neurotoxicity, whereas in compounds **6i–k**, compound **6i**, containing 2-methyl substituent, is the most active congener displaying 100% protection in the scPTZ screen and 16% protection in MES screen at a dose level of 645 and 586 *μ*mol/kg, respectively.

In compounds **6m–p**, compound **6p**, bearing 2,4-dichloro substituents, emerged as the most active drug-like candidate among all the synthesized compounds with 100% protection in the scPTZ screen at a dose level of 636 *μ*mol/kg without any neurotoxicity.

In summary, the synthesized compounds **6a–p** elevate seizure threshold according to their anticonvulsant activity in the scPTZ animal model screen. Additionally, the presence of primary amide group in this type of imidazole-containing arylsemicarbazones is not essential for their anticonvulsant activity in the scPTZ screen.

## 4. Conclusion

Synthesis, characterization, and anticonvulsant activity (phase I) of new imidazole-containing arylsemicarbazones **6a–p** have been successfully achieved. Compounds **6a–p** exhibited anticonvulsant activity principally in the scPTZ screen with compound **6p** which emerged as the most active congener at a dose level of 636 *μ*mol/kg without any neurotoxicity. Moreover, the evoked anticonvulsant activity of the title compounds **6a–p** in the scPTZ screen does not necessitate the presence of a primary amidic moiety in their structure. The assigned (*E*)-configuration of the title compounds **6a–p** was confirmed *via* single crystal X-ray structure of compound **6g**.

## Figures and Tables

**Table 1:** Anticonvulsant potential and neurotoxicity of the target compounds **6a–p**.

Compound no.	Dose^a^ (*μ*mol/kg)	Anticonvulsant activity (% protection)		Neurotoxicity^b^
		scPTZ	MES	
**6a**	750	16	—	0/6
**6b**	667	50	66^c^	0/6
**6c**	680	50	—	2/6
**6d**	718	100	16	0/6
**6e**	718	83	50	0/6
**6f**	684	100	—^c^	0/6
**6g**	972	50	—	0/6
**6h**	616	83	16^c^	0/6
**6i**	645	100	16^c^	0/6
**6j**	586	66	16	0/6
**6k**	520	50	16	1/6
**6l**	818	100	16^c^	0/6
**6m**	565	16	16	2/6
**6n**	814	100	—^c^	0/6
**6o**	662	66	16	0/6
**6p**	636	100	—^c^	0/6
Phenytoin	159	—	100	ND
Phenobarbitone	108	100	—	ND

^a^Doses were administered i.p. ^b^Rotarod test: number of animals exhibiting toxicity/number of animals tested.

^c^Administered dose was 250 mg/kg. Animals (*n* = 6) were examined at 0.5 hr after administration of the test compounds. The dash (—) indicates an absence of anticonvulsant activity at the administered dose. ND: Not determined. The figures in the table indicate the dose whereby the best % protection from seizures was demonstrated.

## Additional Materials

Crystallographic data have been deposited with the Cambridge Crystallographic Data Center (supplementary publication number CCDC-945587). Copies of the data may be obtained free of charge from the Director, CCDC, 12 Union Road, Cambridge, UK.
